# Omentopexy during laparoscopic sleeve gastrectomy: Is it effective in reducing postoperative gastrointestinal symptoms. A retrospective cohort study

**DOI:** 10.1016/j.amsu.2021.102369

**Published:** 2021-04-30

**Authors:** Mohannad AlHaddad, Abrar A. AlAtwan, Talal AlKhadher, Ali AlJewaied, Iman Qadhi, Salman K. AlSabah

**Affiliations:** a Jaber Al Ahmad Al Sabah Hospital, South Surra, Kuwait; b Mubarak Al Kabeer Hospital, Jabriya, Hawalli, Kuwait; c Al Amiri Hospital, Kuwait City, Kuwait; d Faculty of Medicine, Kuwait University, Jabriya, Kuwait

**Keywords:** Gastrointestinal symptoms, Laparoscopic, Sleeve gastrectomy, Nausea, Omentopexy, Vomiting

## Abstract

**Background:**

Postoperative gastrointestinal symptoms are common in patients undergoing sleeve gastrectomy. This study is aimed to assess the effectiveness of omentopexy during laparoscopic sleeve gastrectomy in reducing gastrointestinal symptoms.

**Methods:**

A retrospective analysis of patients who underwent laparoscopic sleeve gastrectomy with and without omentopexy in the period between January 2016 to September 2017. All procedures were performed by three surgeons utilizing the same surgical technique. Data extracted included patient socio-demographics’, preoperative body mass index (BMI), hospitalization period, treatments and post-operative gastrointestinal symptoms. It contained the GERD-Health Related Quality of Life Questionnaire (GERD-HRQL) measuring symptom severity in gastro esophageal reflux disease (GERD). Data were analyzed at 6, 12 and 18 months with reference to weight loss.

**Results:**

A total of 140 patients were included in this study, 70 in each group arm. Age, preoperative BMI, pre-operative co-morbid conditions like hypertension, diabetes, and asthma were considered as confounding variables among the two groups. None of the previous factors were statistically significantly different among both groups. The outcomes of both groups were compared in terms of postoperative nausea, vomiting, regurgitation, intra-hospital stay, medication use, early return to work, and EWL%. None of the previous outcomes except for days of hospital stay and ondansetron use was found to be significantly different between both groups.

**Conclusion:**

Omentopexy does not change the outcome for laparoscopic sleeve gastrectomy in terms of gastrointestinal symptoms or weight loss results.

## Highlights

• Patients undergoing sleeve gastrectomy experience a significant amount of gastrointestinal symptoms postoperatively.
• There are hypothesis that omentopexy reduces post-operative gastrointestinal symptoms.
• Our study indicate that omentopexy during laparoscopic sleeve gastrectomy has no effect in terms of gastrointestinal symptoms or weight loss.

## 1 Background

Metabolic surgery or bariatric surgery first emerged in the 1950's as a method of treatment for severe dyslipidaemias. Since then, the prevalence of obesity, which is now considered a global epidemic, has led the way to recent advancements in bariatric surgery making it a relatively safe procedure and the only clinically evidence-based modality of substantial and sustained weight loss in patients with morbid obesity. Bariatric surgery not only induces weight loss; recent studies have demonstrated not only positive outcomes but the remission of comorbidities associated with obesity including diabetes, hypertension, and dyslipidemia [1,2].

Sleeve gastrectomy was initially part of the duodenal switch procedure, a two stage procedure for the management of high risk patients with morbid obesity, however after proving to be effective in inducing weight loss, it is has been performed as a stand-alone procedure having both restrictive and malabsorptive properties. Sleeve gastrectomy, albeit a bariatric procedure associated with lower morbidity rates comparatively, it has numerous post-operative complications both acute and chronic. The perioperative and postoperative mortality rates are 0.29% and 0.34%, respectively, while the complication rate is 13% [3,4]. The majority of complications associated with LSG occur in the late postoperative period, of which gastrointestinal complaints such as food intolerance and regurgitation, nausea and vomiting, are common. In a conference consensus, between 5 and 22% of patients post LSG suffered from GERD, of those a small percentage converted to another bariatric procedure (typically RYGB) due to the severity of the symptoms [5].

Omentopexy during LSG, is one method amongst others that has been hypothesized to reduce the various complications LSG has been associated with, for example, gastric leaks, gastric obstruction due to strictures or rotation, and GI complaints. A consensus has not been reached currently regarding this hypothesis, as current studies show mixed results, some favorable, while others no significant outcome [6,7]^.^ Contemporary studies have postulated that these debilitating post-operative complications, namely GERD and food intolerance, may be a result of the malpositioning of the gastric sleeve because of the loss of the fixation of the greater curvature of the stomach [8].In light of this hypothesis, this study aims to assess the effectiveness of omentopexy during laparoscopic sleeve gastrectomy in reducing gastrointestinal symptoms.

## 2 Methods

### 2.1 Subjects

This retrospective study was conducted among 140 people with obesity that underwent laparoscopic sleeve gastrectomy at our institution between January 2016 and November 2017. The procedures were performed by three surgeons. Exclusion criteria included incomplete patient information and patients under the age of 17. The entire patient population was extracted from the same general population. Two groups were created, 70 in each arm corresponding to whether or not omentopexy was performed. Ethical approval to conduct the study was obtained from the Ministry of Health and Kuwait Institute for Medical Specialization Ethical Approval Board.

This study was registered at https://www.researchregistry. com/(unique identifying number: researchregistry6628).

Work was reported according to STROCSS criteria [21].

### 2.2 The study tool

Data extracted included demographics, pre-operative comorbid conditions, post-operative gastrointestinal symptoms, total amounts of antinausea medications administered. The days of hospital stay and the days till return to work were also recorded. To measure the symptoms severity in gastroesophageal reflux disease (GERD) a validated GERD-Health Related Quality of Life Questionnaire (GERD-HRQL) was used. GERD-HRQL questionnaire was filled by telephone interviews. Patient who were unwilling to participate or we could not interview were excluded. The scale has 15 questions, which focus on heartburn symptoms, dysphagia, medication effects, and regurgitation. Each question has a scale from 0 to 5, where the higher score defines a better QOL. It also includes an extra question that states whether the patient is currently satisfied, neutral or dissatisfied with their condition. All the above data were extracted at 1 time point however in all patients’ body weight, BMI, and percent excess weight loss (EWL) were examined preoperatively and at 3, 6, and 12 months.

### 2.3 Surgical procedures

After prophylactic antibiotics and under general anesthesia, elastic compression stockings are placed on the legs. Initially the patient is placed in supine position and then when the ports are placed in Trendelenberg. We used a five-port technique which includes a 5 mm port in the epigastrium for liver retraction, a 10 mm supraumbilical slightly to the left for the cameraman, 12 mm right hypochondrial midclavicular (right working port), 15 mm left hypochondrial midclavicular (left working port), and a 5 mm left anterior axillary line port for the assistant's right hand. Using the hook, window in the omental bursa is made approximately 4 cm proximal to the pylorus and then we dissect the greater omentum and the short gastric vessels in close proximity to the gastric serosa up to angle of His using a Ligasure. After dissection, the anesthesiologist passes a 32 French orogastric bougie adjacent to the pylorus. We started by using a 60 mm green or black cartridge stapler firing 2–3 cm proximal to the pylorus and continue firing till reaching the angle of His. The additional hemostasis that may be provided can be offset by staple line reinforcement with buttress material.

**Group 1:** Compromising 70 patients and underwent classic LSG.

**Fig. 1:**
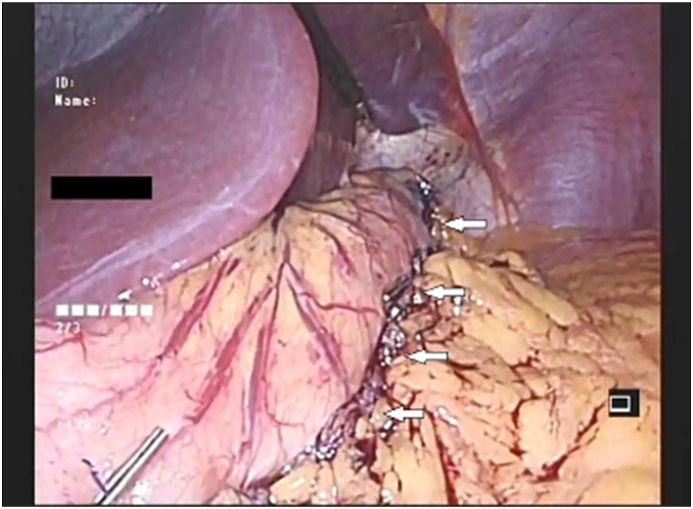
The image delineates a laparoscopic sleeve gastrectomy with omentopexy. The arrows point to site of the omentopexy along the Staple line.

**Group 2:** Compromising 70 patients and underwent LSG with omentopexy. After securing haemostasis to the suture line, we identify the divided free edge of the omentum and suture it to the staple line of the sleeved stomach. Using PDS2-0 starting 2 cm distal to the gastroesophageal junction leaving 1 cm distance between each suture line all the way to the distal part of the sleeved stomach approximately 2 cm proximal to the pylorus (Fig. 1).

### 2.4 Statistical analysis

Continuous variables are expressed as mean and SD. Categorical variables are expressed as frequencies and percentages. Chi square and fisher's exact test were used for testing significance between categorical variables while independent *t*-test were used for generating values for continuous data. Statistical significance was set at a p value less than 0.05. All statistical analyses were performed using SPSS version 25.0.

## 3 Results

The mean age was 37.4 ± 10.9 and 33.7 ± 10.4 for group 1 and group 2, respectively. Male sex represents 26 (37.1%) cases and 9 (12.9%) cases, while the female sex was 44 (62.9%) patients and 61 (87.1%) patients of group 1 and 2, respectively. The mean preoperative BMI in group 1 was 45.1 ± 7.3, while it was 42.5 ± 11.3 in group 2. Among the two groups hypertension constitutes, respectively, 30% (21 cases) and 15.7% (11 cases). Eleven (15.7%) patients of group 1 and 2 had Diabetes. Asthma was found in 12 (17.1%) patients of group 1 and 11 (15.7%) patients of group 2 (Table 1).

**Table 1:** Patients characteristics in the two groups.

N = 70	Group 1 Without Omentopexy N (%)	Group 2 With Omentopexy N (%)	P-Value
Pre-op patient characteristics			
Age (Mean ± Std)	37.4 ± 10.9	33.7 ± 10.4	.279
Range	18–65	17–65	
Gender			.001
Males	26 (37.1%)	9 (12.9%)	
Females	44 (62.9%)	61 (87.1%)	
BMI	45.1 ± 7.3	42.5 ± 11.3	.569
Pre-op comorbid conditions			
HTN	21 (30%)	11 (15.7%)	.485
DM	11 (15.7%)	11 (15.7%)	1
Asthma	12 (17.1%)	11 (15.7%)	1
Surgical History	34 (48.6%)	33 (47.1%)	.000

BMI, body mass index.

The postoperative and complications data is listed in Table 2. There is no significant difference in intra-hospital nausea between the two groups; however the LSG without omentopexy group required significantly longer days of hospital stay and more ondansetron than the LSG with omentopexy. There was no significant difference in days until return to work or the nausea, vomiting, and regurgitation after the patient was discharged between the two groups. There were no intraoperative complications. One patient developed postoperative bleeding in the LSG without omentopexy group. Staple line leakage occurred in one case of the LSG with omentopexy group.

**Table 2:** Postoperative and complications data.

N = 70		Group 1 Without Omentopexy N (%)	Group 2 With Omentopexy N (%)	P- value
Postoperative data				
Intra-hospital Nausea		36 (51.4%)	30 (42.9%)	.084
Days of Hospital Stay(Mean ± Std)		4.5 ± 1.2	1.8 ± 0.97	0.000
Postoperative antinausea pharmacologic requirement				
ondansetron		54 (77.1%)	8(11.4%)	0.000
After Discharge				
Days till return to work(Mean ± Std)	14.6 ± 7.7		11.3 ± 7.9	.665
≤7	12 (17.1%)		31 (44.3%)	
8–14	46 (65.7%)		30 (42.9%)	
≥15	12 (17.1%)		9 (12.9%)	
Nausea	13 (18.6%)		8 (11.4%)	0.344
Vomiting	8 (11.4%)		6 (8.6%)	0.779
Regurgitation	33 (47.1%)		18 (25.7%)	0.790
Morbidity data				
Intraoperative Complications	0(0.0%)		0(0.0%)	1
Postoperative complications				
Bleeding	1(1.4%)		0(0.0%)	1
Leak	0(0.0%)		1(1.4%)	1

### 3.1 Symptoms survey

**Fig. 2:**
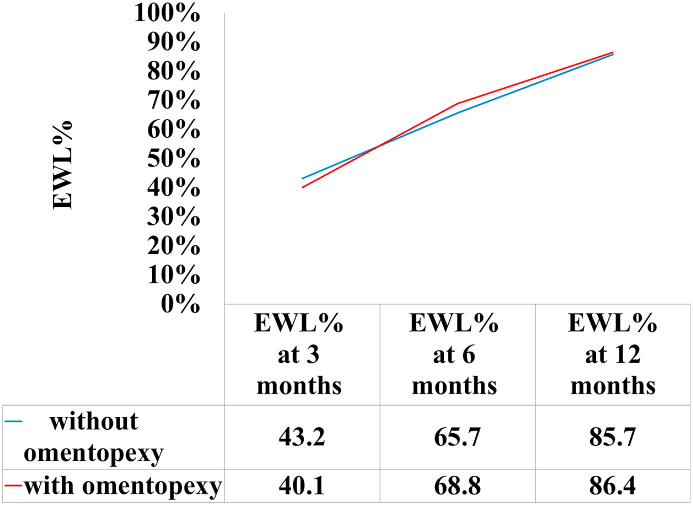
The x-axis represents the postoperative month, while the y-axis represents the percent change in EWL%. There was no significant difference (P < 0.05) at any time point postoperatively.

According to the GERD-Health Related Quality of Life Questionnaire (GERD-HRQL), there was no significant difference in the total score, heartburn score, and regurgitation score between the two groups (Table 3). Overall EWL percentage decreases were not significantly different at any of the measured time points (Fig. 2).

**Table 3:** GERD Health Related Quality of Life Questionnaire scores.

	Group 1 Without Omentopexy N (%)	Group 2 With Omentopexy N (%)	P- value
Total Score			
0	37 (52.9%)	38 (54.3%)	.660
≥1	33 (47.1%)	32 (45.7%)	
Heartburn Score			
0	46 (65.7%)	46 (65.7%)	.682
≥1	24 (34.3%)	24 (34.3%)	
Regurgitation Score			
0	55 (78.6%)	58 (82.9%)	1
≥1	15 (21.4%)	12 (17.1%)

## 4 Discussion

The results obtained from this study suggest that LSG with omentopexy does not effectively reduce gastrointestinal symptoms. Early gastrointestinal symptoms such as nausea and vomiting and regurgitation were experienced similarly by both groups of patients, those that underwent omentopexy during LSG and those that did not. This is further reflected in the lack of significant difference of Heartburn and Regurgitation scores between the two groups. Our study also explored the influence of omentopexy on weight loss; however EWL percentages at 3, 6, and 12 months post LSG with omentopexy paralleled those of the other group.

Various surgical specialties utilize the omentum in their respective practices. The omentum, due to its intrinsic properties, not only promotes healing in the setting of inflammation, it also has the ability to control hemorrhage through pressure and the acceleration of the formation of fibrin clots [9]. Additionally, it has been demonstrated that the omentum has a role in heart repair following myocardial infarction due to its capability of angiogenesis and smooth muscle and endothelium production [10]. In a recent study, Cao et al. have concluded that laparoscopic peritoneal dialysis catheter insertion using omentopexy decreases catheter obstruction and migration [11].

The use of the omentum in LSG in the form of omentopexy entails the suturing of the free end of the greater omentum to the gastric suture line on the greater curvature of the stomach. A number of studies have suggested that this technique may reduce post-operative complications morbidities associated with LSG. One such complication is gastric volvulus, which can occur due to the dissection of the ligaments fixing the posterior gastric wall. A retrospective study in Turkey concluded that omentopexy can in fact prevent gastric volvulus by reducing the mobility and restoring the anatomic position of the stomach post LSG [12]. Gastric leak is another dreaded complication of LSG; theoretically it is plausible that omentopexy should prevent it. However, some studies do claim it to be true, while others; including Hanna et al. state that omentopexy has no role in gastric leak prevention [13].

In addition, the LSG procedure has been suggested in literature to precipitate and aggravate GERD symptoms in patients post operatively. De Groote et al. [14] performed a systematic review in which they compared various bariatric procedures and their effect on GERD. Roux-en-Y gastric bypass was associated with a notable decrease in GERD symptoms while sleeve gastrectomy; however, there was either no change in GERD symptoms or an increase in incidence of GERD.

There are several theories that have been proposed as to reasons why LSG may promote GERD. These include hypotensive lower esophageal sphincter, disruption of the angle of His, reduced gastric compliance with higher intra-gastric pressure, decreased gastric emptying, and the hiatal hernia [15].Taking this into consideration, omentopexy does not correct the previously mentioned and has been shown not to have any effect on the lower esophageal sphincter [16], this may explain the similar GERD scores between patients in both group 1 and 2 in our study (Table 3).

Currently, omentopexy and its association in the reduction of GI symptoms post LSG is still controversial. However, there is increasing evidence supporting the key role of the surgical technique on the incidence of postoperative GERD and other gastrointestinal symptoms. Main surgical technical issues studied are: mid portion narrowing of the sleeve, the redundancy of the upper part of the sleeve, and concomitant hiatal hernia [17]. In addition, the careful dissection of the angle of His while maintaining a safe distance from the gastro-esophageal junction is important in preventing GERD.

In a study by Deas et al. [17], endoscopy was performed in all patients who developed severe reflux symptoms, food intolerance, and vomiting or nausea post LSG. Endoscopic findings were narrowing of the sleeve as a result of excessive tension on the lesser curvature of the stomach when attempting to place the bougie closer to the lesser curvature during the first or second firing of the stapler. There was also partial obstruction of the lower end of the stomach as a result of torsion of the sleeve, which could be prevented by maintaining the same distance between the lesser curvature and the entire staple line during division of the stomach.

The dose-response association between weight and GERD has been proven in numerous studies. It has been reported that the prevalence of GERD has been shown to increase with increasing BMI [18,19].Thus it can be depicted that weight loss should theoretically alleviate symptoms of GERD. Results of a prospective study in the United States (US) showed that weight loss led to a significant improvement in GERD symptoms, and they established weight loss as an important factor in the treatment of GERD [20].The fact that weight loss or EWL% were almost identical in both groups 1 and 2 may play a factor in the lack of difference in GERD scores, as omentopexy may have no role in weight loss.

## 5 Limitations

Limitations of the research include the small sample size; therefore further larger multicentric studies are needed to be performed to confirm our results. Additionally, this is a retrospective study so it lacks randomization. RCT studies on this point should be conducted in order to achieve a more potent scientific level.

## 6 Conclusion

Omentopexy may not change the outcome for laparoscopic sleeve gastrectomy in terms of gastrointestinal symptoms or weight loss results. Currently, the role of omentopexy is controversial and further detailed studies need to be performed in validating the role of omentopexy. It can be suggested that surgical technique is one of the main factors in the promotion of gastrointestinal symptoms and GERD, and hence correction of technical errors may be the best method in their prevention.

## Declaration of competing interest

The authors declare that they have no competing interests.

## Registration of research studies

1.Name of the registry: http://www.researchregistry.com
2.Unique Identifying number or registration ID: researchregistry6628
3.Hyperlink to your specific registration (must be publicly accessible and will be checked):
